# Epidemiology and risk factors for multi-drug resistant hospital-acquired urinary tract infection in patients with liver cirrhosis: single center experience in Serbia

**DOI:** 10.1186/s12879-019-3761-5

**Published:** 2019-02-12

**Authors:** Tamara Milovanovic, Igor Dumic, Jelena Veličkovic, Milica Stojkovic Lalosevic, Vladimir Nikolic, Ivan Palibrk

**Affiliations:** 1 School of Medicine, University of Belgrade, Belgrade, Serbia; 2 Department of Gastroenterology and Hepatology, Clinical Center of Serbia, Belgrade, Serbia; 3 Department of Hospital Medicine, Mayo Clinic Health System, Eau Claire, WI USA; 4 Mayo Clinic College of Medicine and Science, Rochester, MN USA; 5 Department of Anesthesiology, Clinical Center of Serbia, Belgrade, Serbia

**Keywords:** Liver cirrhosis, Urinary tract infection, Multi-drug resistant organism, Immune dysfunction

## Abstract

**Background:**

Cirrhosis-associated immune dysfunction syndrome (CAIDS) has been identified in patients with liver cirrhosis (LC), predisposing them to a wide variety of infections. In patients with LC, healthcare-associated infections involving multi-drug resistant (MDR) bacteria have increased significantly over the last decades. Among them, hospital-acquired urinary tract infections (HA-UTI) are the most common. This study aimed to investigate the rates of antimicrobial resistance among patients with LC and HA-UTI and to determine risk factors associated with their development among patients hospitalized in tertiary care facility in Serbia.

**Methods:**

This retrospective study included 65 hospitalized patients with LC who had developed HA-UTI. We examined the epidemiology of these infections concerning resistance to the most commonly used antimicrobials and patient-specific risk factors associated with HA-UTI development by MDR pathogens.

**Results:**

The most frequently isolated organisms were *Enterococcus* spp. (*n* = 34, 52.3%), *Klebsiella* spp. (*n* = 10, 15.4%), and *E.coli* (*n* = 6, 9.2%). Thirty-five isolates (53.8%) were identified as MDR, and 30 (46.2%) were non-MDR.We found a statistically significant difference in the distribution of MDR and non-MDR strains, based on Gram staining, with the majority of Gram-negative pathogens being MDR (*p* = 0.005). We identified age ≥ 65 years (*p* = 0.007), previous use of cephalosporins as empiric therapy (*p* = 0.042), and the presence of hepatic encephalopathy (*p* = 0.011) as independent risk factors for the development of MDR UTIs.

**Conclusion:**

This is the first study from Serbia and the Balkans concerning the changing epidemiology of MDR UTI in patients with LC. Our study showed that more than half of HA-UTI was caused by MDR and the most common pathogen was *Enterococcus spp.* The overall resistance to ceftriaxone was 92%. Our findings underscore the need for institutions to individualize protocols for treatment of hospital-acquired infections, particularly in immunocompromised populations.

## Background

In community and hospital settings, urinary tract infections (UTIs) are one of the most commonly encountered infections. Clinically, UTIs can be uncomplicated or complicated. Uncomplicated UTIs affect otherwise healthy individuals with no structural abnormalities of the urinary tract. In contrast, complicated UTIs occur in patients with structural urinary tract abnormalities, immunosuppression, or are associated with indwelling urinary catheters [1]. Hospital-acquired (HA) or nosocomial UTIs (HA-UTIs) account for nearly 40% of all HA infections [2, 3]. An HA-UTI is defined as an infection that occurs in a patient during hospitalization, or care in any other healthcare facility, which had not been present nor incubating at the time of admission [3].

The most common laboratory criterion used for defining significant bacteriuria is the presence of ≥10^5^ colony forming units (CFU) per milliliter of urine [4]. However, a significant number of patients (between 30 and 50%) with acute urethral syndrome have colony counts of <10^5^CFU/mL [4]. For this reason, many laboratories, including the laboratory at our institution, have opted to use lower colony counts of >10^4^CFU/mL as a criterion for interpreting and reporting results [4].

Patients with liver cirrhosis (LC) have an altered immune system that predisposes them to a wide variety of infections. Cirrhosis-associated immune dysfunction syndrome (CAIDS) results from overwhelming activation of pro-inflammatory cytokines in cirrhosis and portosystemic shunting that leads to a decrease in cytokines, endotoxins, and bacterial clearance via the portal circulation of the liver [5].

All the systemic inflammatory response syndrome (SIRS) components in LC are impaired, significantly contributing to the development of infection. Infection increases mortality in patients with LC 4-fold compared to the general population [5–7]. Delayed intestinal transit time, bacterial overgrowth, increase in pro-inflammatory cytokines and nitric oxide, as well as portosystemic shunting, all contribute to increased translocation of bacteria into mesenteric lymph nodes, ascites, and systemic circulation in patients with decompensated LC [5]. The most common infections in patients with LC are spontaneous bacterial peritonitis (SBP) (25–31%), UTI (20–25%), and pneumonia (15–21%), while a combination of bacteremia and soft tissue infection represent the remaining 23% [6, 7]. A reduced number of liver reticuloendothelial (RE) cells, a dysfunctional increase in monocyte and neutrophil activation, and a decrease in bacterial phagocytosis in patients with LC further impair clearance of bacteria, endotoxins, and cytokines from the circulation [5, 6]. The diminished phagocytic activity in LC combined with the decrease in bactericidal and opsonization capacity is also associated with lower levels of immunoglobulins (Ig) IgM, IgG, and IgA, and with C3, C4, and CH50 concentrations in ascites. The immunocompromised state in patients with LC is further complicated with malnutrition, the use of immunosuppressive medications, and alcohol consumption, leading to a decrease in T and B cells and natural killer cells.

The highest risks of developing infection pertain to hospitalized patients with LC who develop gastrointestinal (GI) bleeding, hepatic encephalopathy, and renal failure [7]. These patients frequently have infections that are resistant to multiple antibiotics, leading to a worse outcome [5, 7]. UTI can present in various forms, from uncomplicated cystitis to complicated pyelonephritis leading to sepsis (42–65%); hence, it is essential to prevent these infections, to recognize them early in the clinical course, and to manage them appropriately to reduce morbidity and mortality [7].

The primary causative bacterial uropathogens are Gram-negative bacilli such as *Escherichia coli (E.coli)* and *Klebsiella* spp.*,* while Gram-positive bacteria such as *Enterococci* and *Staphylococcus aureus* contribute to approximately12–20% of infections [7, 8]. The prevalence of multi-drug resistant (MDR) organisms in patients with LC has been increasing over the last decade, especially in healthcare settings [7–9].

An MDR strain of bacteria is defined as in vitro resistance to at least one agent in three or more antimicrobial categories [8]. The main risk factors for developing an MDR bacterial infection in patients with LC are current or recent hospitalization, long-term norfloxacin prophylaxis, use of systematic antibiotics within the previous 30 days, upper GI bleeding, and diabetes mellitus (DM) [9]. Due to significant differences in the definition of differing levels of bacterial resistance, an international group of experts have proposed a new classification for bacterial resistance, as follows: MDR bacteria, if resistant to at least one agent in three or more antimicrobial classes; Extensively Drug Resistant (XDR) bacteria, if only sensitive to agents from one or two different classes of antibiotics, and Pan-drug Resistant (PDR) bacteria, if resistant to all agents in all tested antibiotic classes [10, 11].

Our study aimed to assess the local epidemiology and antimicrobial resistance rates among pathogens isolated from patients with decompensated LC who developed UTI in healthcare settings. Also, we wanted to determine the extent of empiric antibiotic therapy failure and analyze the patients’ specific characteristics that significantly correlate with the acquisition of infection with MDR strains.

## Methods

### Patients

This retrospective study was conducted at a tertiary care facility within a university teaching hospital, in the Department of Gastroenterology and Hepatology at Clinical Center, in Belgrade, Serbia. The study comprised 65 consecutively hospitalized patients, between 2013 and 2016, who had an initial diagnosis of LC and who were subsequently diagnosed with an HA-UTI. Exclusion criteria were as follows: patients aged< 18 years, pregnancy, presence of hepatocellular carcinoma, previous transplantation, treatment with immunosuppressive agents, and human immunodeficiency virus infection.

We collected demographic, laboratory, and clinical data, including potential risk factors (such as the recent use of antibiotics, hospitalization within 90 days prior to current hospitalization, DM, and the presence of a urinary catheter) and comorbidities.

According to age at the time of hospitalization and UTI development, all patients were stratified into two age groups: Group 1 comprised patients aged between 35 and 64 years, and group 2 comprised patients ≥65 years.

### Severity of LC

LC severity was assessed using the Child-Pugh Score, the Model of End-Stage Liver Disease (MELD) score, and the CLIF Consortium Acute Decompensation score (CLIF-C ADs) [1, 8, 12].

### Diagnosis of UTI

A UTI diagnosis was made according to the following clinical criteria: symptoms suggestive of UTI including suprapubic tenderness and/or costovertebral angle tenderness and/or increased urinary frequency, urgency, or dysuria with or without fever (> 38.0 °C), with a confirmatory urine leukocyte count of 15 cells or higher per high-power field, and a positive urine culture with mono-bacterial growth ≥10,000 CFU/mL. Patients with polymicrobial infection were included only if both isolated species exhibited a growth of ≥10,000 CFU/mL on urine culture [2, 9].

Urine samples were obtained using the clean-catch midstream technique following cleansing of the foreskin and mucous membranes adjacent to the urethral orifice before micturition. A straight catheter technique was used for patients who could not provide urine using the clean-catch midstream technique.

The Kirby-Bauer disk diffusion method was used to perform microbial susceptibility testing (MST), according to the Clinical and Laboratory Standards Institute (CLSI) guidelines [13, 14]. An automated plate reader distinguished treatment effects after only six hours of incubation. Both intermediate and resistant strains were classified as resistant. Rates of antimicrobial resistance were defined as: low (< 10%), moderate (10–20%) and high (> 20%) [15].

### Ethics approval and consent to participate

This study was conducted following the approval of the Ethics Committee of the Clinical Center of Serbia, and in accordance with the Helsinki Declaration. As this was a retrospective study, patient consent was not deemed necessary according to the IRB committee at our institution.

### Empirical antibiotic treatment in cirrhosis

According to general guidelines and hospital protocol patients with LC and with a history of GI bleeding or previous SBP were treated with antibiotic therapy. Additionally, treatment with broad-spectrum antibiotics was used when an infection was suspected following collection of the culture specimens [16]. Empiric antibiotic treatment was considered appropriate and applicable only when isolated bacteria were found to have an in vitro susceptibility to a particular antibiotic.

### Treatment failure

Empiric therapy failure was defined as persistent or worsening UTI symptoms despite antimicrobial therapy.

### Classification of multi-resistant bacteria

The European Center for Disease Prevention and Control (ECDC) definitions for MDR bacteria were used [10]. According to these international guidelines in regard to differing degrees of MDR, infections were classified as: (1) MDR, (2) XDR and, (3) PDR [10, 11]. Antimicrobial agents analyzed in our study included the following: penicillin; penicillin with beta-lactamase inhibitors; aminoglycosides; anti-pseudomonal penicillin; carbapenems; cephalosporins, including extended spectrum cephalosporins; fluoroquinolones; folate pathway inhibitors; glycopeptides and glycylcyclines.

### Statistical analysis

Data are presented as mean ± SD or median (interquartile range [IQR]) for continuous variables, depending on normality of data distribution, and number (percentage) for categorical variables. Normality was tested using the Shapiro-Wilk test. Clinical and demographic characteristics of patients with MDR and non-MDR infections were compared and analyzed using the independent samples t-test or the Mann-Whitney U test for continuous variables. A Chi-square or Fisher’s exact test was used for analysis of categorical data, where appropriate. All tests were two-tailed and a *p*-value< 0.05 indicated statistical significance.

Logistic regression analysis was performed to identify the independent predictors of MDR UTIs. Factors with a *p*-value < 0.15 in the univariate analysis were included in a multivariate logistic regression model. Logistic regression analysis was performed to identify the independent predictors of MDR UTIs. Independent variables in the final multivariable model were selected using the forward stepwise method. The univariate analysis was used only as an intermediate step to find the most appropriate variables for the multivariate analysis. The collinearity and interaction between variables were assessed in the final model and the adjusted odds ratios (OR) and corresponding 95% confidence intervals (CI) for independent risk factors were calculated. The overall robustness of the model was assessed using the Hosmer-Lemeshow goodness-of-fit test. Statistical analyses were performed with SPSS 19.0 software (SPSS Inc., Chicago, IL,USA).

## Results

### Patients

A total of 65 patients with LC and HA-UTI were included in the study. The mean age was 60.8 ± 11.0 years (range, 39–84 years), and 48 (73.8%) were male. Alcohol abuse (*n* = 47, 72.3%), autoimmune (*n* = 7, 10.8%), viral (*n* = 6, 9.2%), metabolic (*n* = 2, 3.1%), and cryptogenic (*n* = 3, 4.6%) etiologies of LC were identified. No patients had overlapping etiology. Patient demographic data are shown in Table 1.

**Table 1:** Clinical and demographic characteristics of patients with MDR and non-MDR infections

Variable	MDR (*n* = 35)	Non-MDR (*n* = 30)	*P*
Male	27 (56.3)	21 (43.8)	0.579
Age	63.8 ± 11.9	57.3 ± 9.7	**0.018**
Age groups			
35–64	17 (41.4)	24 (58.6)	0.214
> 65	18 (75.0)	6 (25.0)	**0.011**
Etiology of cirrhosis			
Alcohol	27 (57.4)	20 (42.6)	0.411
Viral	4 (66.7)	2 (33.3)	0.678
Metabolic	1 (50.0)	1 (50.0)	1.000
Autoimmune	**1 (14.3)**	**6 (85.7)**	**0.026**
Cryptogenic	2 (66.7)	1 (33.3)	1.000
*Comorbidities*			
DM	10 (66.7)	5 (33.3)	0.377
Renal Insufficiency	3 (60.0)	2 (40.0)	1.000
Renal or urethral structural abnormalities	3 (50.0)	3 (50.0)	1.000
Co-infections			
Pneumonia	10 (66.7)	5 (33.3)	0.377
Sepsis	3 (60.0)	2 (40.0)	1.000
*Clostridium difficile*	3 (75.0)	1 (25.0)	0.618
Clinical characteristics			
Indwelling Urinary Catheter	15 (45.5)	18 (54.5)	0.216
Antibiotic use in the last 7 days	26 (65.0)	14 (35.0)	**0.040**
Antibiotic use in the last 90 days	3 (50.0)	3 (50.0)	1.000
Fluoroquinolones	9 (50.0)	9 (50.0)	0.784
Cephalosporins	12 (80.0)	3 (20.0)	**0.021**
Aminoglycosides	2 (40.0)	3 (60.0)	0.655
Metronidazole	14 (70.0)	6 (30.0)	0.108
Other	5 (55.6)	4 (44.4)	1.000
CP Class C	27 (61.4)	17 (38.6)	0.111
MELD score	21.8 (6.65)	21.9 (5.42)	0.913
CLIF-C AD score	90.1 (9.1)	86.2 (11.2)	0.126
Ascites (1, 2, 3)	30 (54.5)	25 (45.5)	1.000
Encephalopathy (1, 2, 3)	22 (68.8)	10 (31.3)	**0.025**
Hepatorenal syndrome	3 (50.0)	3 (50.0)	1.000
History of variceal hemorrhage	5 (45.5)	6 (54.5)	0.742
BUN (mmol/l)	14.5 [10.2]	9.7 [6.1]	**0.028**
Creatinine (μmol/l)	100.5 [71.5]	91.9 [61.8]	0.609
Serum sodium (mmol/l)	131.9 [6.7]	132.7 [7.5]	0.611
Ferritin (μg/l)	611.4 [360.8]	169.2 [395.7]	**0.024**
Billirubin (μmol/l)	33.0 [82.7]	49.1[136.1]	0.490
Outcomes			
Length of Hospitalization	26,0 [30.0]	27.5 [19.0]	0.980
Change of therapy	19 (59.4)	13 (40.6)	0.459
Death	9 (64.3)	5 (35.7)	0.546

Notes: This table shows clinical and demographic characteristics of patients with MDR and non MDR infections. The sample size is 65 patients. Descriptive statistics are presented as means ± SD, medians [IQR] and numbers (%). MDR stands for multidrug resistant; *DM* Diabetes mellitus, *CP* Child-Pugh score, *MELD* Model for End Stage Liver Disease score, *CLIF-C AD* Chronic Liver Failure-Consortium Acute Decompensation score, *BUN* Blood Urea Nitrogen, *AST* Aspartate aminotransferase, *ALT* Alanine aminotransferase and *INR* International normalized ratio

All patients had decompensated LC. Of 65 patients, 21 (32.3%) had a Class B Child-Pugh score, and 44 (67.7%) had a Class C Child-Pugh score, with a mean MELD score of 21.88 ± 6.07, and a mean CLIF-C ADs of 88.34 ± 10.26. Ascites had been diagnosed in 55 (84.6%) patients, 32 (49.9%) patients had hepatic encephalopathy either at admission or during hospitalization, 15 (23%) patients had a diagnosis of DM, and 33 (50.8%) patients had been catheterized.

### Isolated organisms

Isolated pathogens are shown in Table 2.

**Table 2:** Isolated bacterial uropathogens in our cohort of patients

Pathogen	*n*	(%)
*Enterococcus spp*	34	52.3
*Klebsiella spp*	10	15.4
*Escherichia coli*	6	9.2
*Proteus mirabilis*	5	7.7
*Acinetobacter baumanii*	3	4.6
*Pseudomonas aeruginosa*	2	3.1
*Providencia rettgeri*	1	1.5
*Moraxella catarrhalis*	1	1.5
MSSA	1	1.5
MRSA	2	3.1
Total	65	100

Notes: This table presents the most commonly isolated bacterial pathogens in patients with liver cirrhosis and hospital acquired urinary tract infection. MSSA stands for methicillin-sensitive *Staphylococcus aureus,* and *MRSA* Methicillin-resistant *Staphylococcus aureus*

The most frequently isolated organisms were *Enterococcus* spp. (*n* = 34, 52.3%), *Klebsiella* spp. (*n* = 10, 15.4%), *E.coli* (*n* = 6, 9.2%), and *Proteus mirabilis* (*n* = 5, 7.7%). *Acinetobacter baumanii*, *Pseudomonas aeruginosa, Providencia rettgeri*, and *Moraxella catarrhalis* were isolated in 3 (4.6%), 2 (3.1%), 1 (1.5%), and 1 (1.5%) instances, respectively. Methicillin-sensitive *Staphylococcus aureus* (MSSA) was seen on one occasion (1.5%) and methicillin-resistant *Staphylococcus aureus* (MRSA) was isolated twice (3.1%). There were no polymicrobial infections documented.

The distribution of MDR and non-MDR strains among the isolates is shown in Table 3.

**Table 3:** Distribution of MDR and Non-MDR pathogens

Pathogen	MDR *n* (%)	Non-MDR *n* (%)	*P*
*Enterococcus spp*	12 (35.3)	22 (64.7)	0.003
*Enterobacteriacae* ^*a*^	18 (81.1)	4 (18.2)	0.001
Other^b^	5 (55.6)	4 (44.4)	1.000
Gram+/Gram-	14 (37.8) / 21 (75.0)	23 (62.2) / 7 (25.0)	0.005
Total	35 (53.8)	30 (46.2)	NA

Notes: This table reports the distribution of MDR and non MDR uropathogens in our cohort of 65 patients with liver cirrhosis. ^a^ single XDR *Klebsiella spp*, 16 isolates ESBL-*E* (72.7%). ^b^*Pseudomonas aeruginosa*, *Moraxella catarrhalis*, *Acinetobacter baumanii*, methicillin-sensitive *Staphylococcus aureus*, methicillin-resistant *Staphylococcus aureus*

Thirty-five isolates (53.8%) were found to be MDR, and 30 (46.2%) were non-MDR. *Enterococcus* spp. isolates were more likely to be non-MDR (*n* = 22, 64.7%, *p* = 0.003), whereas *Enterobacteriaceae* were mainly MDR strains (*n* = 18, 81.1%, *p* = 0.001). Vancomycin-resistant enterococcus (VRE) was isolated on 7 occasions (20.6%) while 16 of the *Enterobacteriaceae* isolates (72.7%) were extended-spectrum beta-lactamase-producing *Enterobacteriaceae* (ESBL-E). Only one *Klebsiella* strain was XDR, and no PDR pathogens were isolated. There was a statistically significant difference in the distribution of MDR and non-MDR strains based on Gram staining, with the majority of Gram-negative pathogens being MDR (*n* = 21, 75%), and the majority of Gram-positive bacteria predominantly observed in the non-MDR patients (*n* = 23, 62.2%, *p* = 0.005).

### Antibiotic resistance rates

The resistance rates of the 65 isolated pathogens are shown in Table 4.

**Table 4:** Resistance rates of isolated pathogens

*ABs*	*Enterococcus spp* ^b^		*Enterobacteriaceae*		Total^a^		*P value*	Overall
	MDR (*n* = 12)	Non-MDR (*n* = 22)	MDR (*n* = 18)	Non-MDR (*n* = 4)	MDR (*n* = 35)	Non-MDR (*n* = 30)		*n* = 65
AMP	8/11 (72.7)	2/20 (10.0)	16/18(88.9)	4/4 (100)	29/34(85.3)	10/28 (35.7)	0.000	39/62 (62.9)
AMX	8/11 (72.7)	2/20 (10.0)	16/18(88.9)	4/4 (100)	29/34 (85.3)	9/27 (33.3)	0.000	38/61 (62.3)
AMP-SL	1/2 (50.0)	1/6 (50.0)	16/18(88.9)	4/4 (100)	8/12 (66.7)	3/8 (37.5)	0.362	11/20 (55.0)
AM-CL	7/9 (77.8)	2/14 (14.3)	16/18(88.9)	2/4 (50.0)	26/30(86.7)	7/22 (31.8)	0.000	33/52 (63.5)
P-TZ	2/2 (100)	0/2 (0)	5/13 (38.5)	1/3 (33.3)	9/17 (52.9)	1/7 (14.3)	0.172	10/24 (41.7)
MER	4/5 (80.0)	0/4 (0)	6/15 (40.0)	1/3 (33.3)	14/24(58.3)	1/9 (11.1)	0.021	15/33 (45.4)
IMI	6/7 (85.7)	1/6 (16.7)	5/13 (38.5)	0/3 (0)	16/25(64.0)	1/11 (9.1)	0.003	17/36(47.2)
ERT	4/4 (100)	0/4 (0)	8/13 (61.5)	1/2 (50.0)	17/22(77.3)	3/8 (37.5)	0.078	20/30 (66.7)
CFAZ	–	–	16/16 (100)	1/3 (33.3)	32/32 (100)	20/23 (86.9)	0.068	52/55 (94.5)
CEPH	–	–	18/18 (100)	1/4 (25.0)	35/35 (100)	21/25 (84.0)	0.026	56/60 (93.3)
CEFU	–	–	16/16 (100)	1/4 (25.0)	32/32 (100)	21/26 (80.8)	0.014	53/58 (91.4)
CEFO	–	–	16/17(94.1)	1/4 (25.0)	32/33(97.0)	21/25 (84.0)	0.154	53/58 (91.4)
CFTX	–	–	17/17 (100)	1/4 (25.0)	33/33 (100)	21/25 (84.0)	0.030	54/58 (93.1)
CFTA	–	–	18/18 (100)	1/3 (33.3)	33/34(97.0)	20/24 (83.3)	0.004	53/58 (91.4)
CEFP	–	–	18/18 (100)	1/4 (25.0)	33/34(97.0)	20/24 (83.3)	0.149	53/58 (91.4)
AMI	1/1 (100)	1/3 (33.3)	4/16 (25.0)	1/3 (33.3)	8/20 (40.0)	2/10 (20.0)	0.419	10/30 (33.3)
GEN	4/8 (50.0)	3/6 (50.0)	7/13 (53.8)	1/4 (25.0)	14/25(56.0)	4/12 (33.3)	0.295	18/37 (48.6)
CIP	11/11(100)	12/13 (92.3)	10/13(76.9)	1/4 (25.0)	23/26(88.5)	13/19 (68.4)	0.137	36/45 (80.0)
LEVO	1/1 (100)	2/2 (100)	3/3 (100)	1/3 (33.3)	5/5 (100)	3/7 (42.8)	0.081	8/12 (66.7)
VAN	5/12 (41.7)	2/21 (9.5)	1/5 (20.0)	1/1 (100)	7/20 (35.0)	3/23 (13.0)	0.148	10/43 (23.2)
TEI	7/12 (58.3)	1/21 (4.8)	1/5 (20.0)	1/1 (100)	10/19(52.6)	2/22 (9.1)	0.005	12/41 (29.3)
T-SX	1/2 (50.0)	2/4 (50.0)	1/5 (20.0)	1/1 (100)	19/24(79.2)	7/11 (63.6)	0.416	26/35 (74.3)
NIF	6/8 (75.0)	2/6 (33.3)	5/5 (100)	1/2 (50.0)	11/15(73.3)	3/8 (37.5)	0.179	14/23 (60.9)
LIN	1/4 (25.0)	0/5 (0)	1/7 (14.3)	1/1 (100)	1/7 (14.3)	1/7 (14.3)	1.000	2/14 (14.3)
TIG	1/3 (33.3)	0/3 (0)	0/1 (0)	1/2 (50.0)	1/6 (16.7)	1/5 (20.0)	1.000	2/11 (18.2)

Notes: This table shows the resistance rates to most commonly used antibiotics in clinical practice with comparison between MDR and non MDR pathogens. ^b^ due to the intrinsic cephalosporin resistance, data are not shown for enterococci but are included in totals and the overall analysis. ^a^*Acinetobacter baumanii, Pseudomonas aeruginosa*, *Moraxella catarrhalis*, methicillin-sensitive *Staphylococcus aureus*, methicillin-resistant *Staphylococcus aureus* included in analysis but not shown. *ABs* stands for antibiotics; *AMP* Ampicillin, *AMX* Amoxicillin, *AMP-*SL Ampicillin sulbactam, *AM-CL* Amoxicillin-clavulanic acid, *P-TZ* Piperacillin-tazobactam, *MER* Meropenem, *IMI* Imipenem, *ERT* Ertapenem, *CFAZ* Cefazolin, *CEPH* Cephalexin, *CEFU* Cefuroxime, *CEFO* Cefotaxime, *CFTX* Ceftriaxone, *CFTA* Ceftazidime, *CEFP* Cefepime, *AMI* Amikacin, *GEN* Gentamicin, *CIP* Ciprofloxacin, *LEVO* Levofloxacin, *VAN* Vancomycin, *TEI* Teicoplanin, *T-SX* Trimethoprim-sulfamethoxazole, *NIF* Nitrofurantoin, *LIN* Linezolid, and *TIG* Tigecycline

Data are represented based on MST results, and stratified according to the pathogens, MDR vs non-MDR, and overall resistance rates. For each antibiotic, the number of resistant isolates is shown as well as the number of in vitro tests for that agent. Overall, low resistance (< 10%) was not seen against any of the antibiotics that were tested. The overall resistance rates to ceftriaxone, ampicillin-sulbactam, and amoxicillin-clavulanic acid were 93.1, 55.0, and 63.5%, respectively. Similarly, a high resistance against ciprofloxacin and trimethoprim-sulfamethoxazole was detected (80.0 and 74.3%, respectively). The overall resistance against the tested carbapenems was 45.4% for meropenem, 47.2% for imipenem, and 66.7% for ertapenem. Glycopeptides demonstrated a better resistance profile with an overall resistance to vancomycin and teicoplanin of 23.2 and 29.3%, respectively. Resistance to nitrofurantoin, recommended for the treatment of uncomplicated nosocomial UTIs in patients with LC, was 60.9%. Antibiotic resistance rates of MDR pathogens were, as expected, higher than non-MDRs for the majority of tested antibiotics including ampicillin (85.3% vs 35.7%), amoxicillin-clavulanic acid (86.7% vs 31.8%), meropenem (58.3% vs 11.1%), imipenem (64.0% vs 9.1%), and teicoplanin (52.6% vs 9.1%), and the differences were statistically significant. Resistance rates to cephalosporins were extremely high, ranging from 83.3 to 100% and, while statistically significant, the difference between MDR and non-MDR pathogens was clinically irrelevant, due to extremely high resistance in both groups. The most effective antibiotics against *Enterococcus* spp. isolates were linezolid and vancomycin, with resistance rates of 25.0 and 41.7% for MDR, and 0 and 9.5% for the non-MDR strains, respectively. *Enterococcus* spp. showed high resistance rates to almost all other antibiotics, reaching 100% for piperacillin-tazobactam, ertapenem, amikacin, ciprofloxacin, and levofloxacin amongst MDR strains. The pattern of antibiotic resistance in *Enterobacteriaceae* isolates showed the highest resistance to ampicillin (88.9% vs 100%, MDR vs non-MDR, respectively); cephalosporins (ceftriaxone, 100% vs 25%, MDR vs non-MDR, respectively); and nitrofurantoin (100% vs 50%, MDR vs non-MDR, respectively). High resistance rates were seen against the carbapenem group of antibiotics: 40 and 33.3% to meropenem, 38.5 and 0% to imipenem, and 61.5 and 50.0% to ertapenem, for MDR vs non-MDR, respectively.

### Empiric therapy failure and change in therapy

As expected, patients with MDR UTI had a significantly higher empiric therapy failure rate (*p* = 0.039). The failure rate was unknown in 17 patients (26.2%) since the susceptibility of the isolated pathogen was not determined in 11 patients with MDR UTIs and in 6 patients with non-MDR UTIs. When we excluded these patients from the analysis, 15 (62.5%) patients with MDR UTI had therapy failure in comparison to 7 (29.2%) with non-MDR UTI (*p* = 0.02). Although statistically insignificant, a higher proportion of patients who required a change of therapy were found to have an MDR UTI (19, 59.4%, *p* = 0.459).

### Patient characteristics in MDR and non-MDR UTIs

Patient demographic and clinical characteristics according to MDR and non-MDR infection are summarized in Table 1. There was a statistically significant difference in age between the two groups, with older patients and, in particular, those ≥65 years (75%) having MDR UTIs (*p* = 0.018 and *p* = 0.011, respectively). The patients in the two groups did not differ in the etiology of LC, with the exception of the autoimmune etiology patient group, where 85.7% of patients had non-MDR UTI (*p* = 0.026). There were no differences between patients with MDR and non-MDR UTI with respect to comorbidities, co-infections, presence of urinary catheter, severity of liver disease, and outcomes. Exposure to antibiotics 7 days prior to UTI diagnosis was evidenced in 65% of patients with MDR UTI (*p* = 0.040). Furthermore, 80% of patients who had been exposed to cephalosporins in the previous 7 days were in the MDR group (*p* = 0.021). The presence of ascites did not differ between the groups; however, encephalopathy was seen in 68.8% of patients in the MDR group (*p* = 0.025). Regarding biochemical parameters, blood urea nitrogen (BUN) and serum ferritin were both higher in the MDR group (14.5, IQR; 10.2 mmol/L, *p* = 0.028, and 611.4, IQR; 360.8 μg/L, *p* = 0.024, respectively).

### Patient specific risk factors associated with MDR HA-UTI

In univariate analysis, age ≥ 65 years, an autoimmune etiology of LCs, antibiotic use in the previous 7 days, cephalosporin prophylaxis, hepatic encephalopathy, BUN, and serum ferritin were found to be associated with MDR UTI. Multivariate logistic regression with a forward selection was used to identify variables independently associated with MDR UTI. Age ≥ 65 years (OR: 4.23, 95% CI; 1.39–12.89, *p* = 0.007), empiric cephalosporin therapy (OR: 3.61, 95% CI; 1.81–17.24, *p* = 0.04), and hepatic encephalopathy (OR: 4.99, 95% CI; 1.44–17.30, *p* = 0.01) were found to be independent predictors of MDR UTIs in our study (Table 5).

**Table 5:** Risk factors associated with MDR HA-UTI

Risk factor	Univariate analysis		Multivariate analysis	
	OR (95% CI)	*p*	aOR (95% CI)	*p*
Age > 65 years	6.33 (1.66–24.10)	< 0.001	4.23 (1.39–12.89)	**0.007**
Autoimmune etiology of cirrhosis	8.50 (1.39–12.89)	0.006	3.88 (0.82–6.22)	0.08
Antibiotic use in the previous 7 days	3.30 (1.16–9.37)	0.002	2.66 (0.91–3.08)	0.29
Cephalosporin prophylaxis	4.70 (1.18–18.70)	0.001	3.61 (1.81–17.24)	**0.04**
Hepatic encephalopathy	3.38 (1.22–9.41)	< 0.001	4.99 (1.44–17.30)	**0.01**
BUN	1.08 (1.00–1.61)	0.01	1.01 (1.00–1.03)	0.14
Serum ferritin	1.01 (1.01–1.87)	0.04	0.84 (0.47–1.68)	0.62

*This table presents risk factors associated with developement of MDR HA-UTI. MDR* Multi-drug resistant, *HA* Hospital acquired, *UTI* Urinary tract infection, *OR* Odds ratio, *CI* Confidence interval, *aOR* Adjusted odds ratio, *BUN* Blood urea nitrogen

## Discussion

The increase in antimicrobial resistance and the lack of new treatment options for MDR organisms are causing public concern worldwide. Patients with LC due to an immunocompromised state are at an increased risk of developing infections and progressing to sepsis. Despite recent advances in the treatment of sepsis, short-term mortality in this group of patients remains significantly high, at up to 75% [17–19]. UTIs are very common in patients with LC and comprise 40% of hospital-acquired (HA) bacterial infections [5, 20]. Although more frequent among those with LC, the incidence of UTI in this population does not correlate with the severity of liver disease but is associated with sex (females have a higher risk) and DM [6].

The most common bacterial isolates are Gram-negative bacteria with *E. coli*, in the majority of patients. Thus far, treatment of UTI with quinolones has been effective in approximately 95% of patients [6].

Multi-drug resistant HA infections, including UTIs, are being increasingly reported, especially in southern Europe [5, 21, 22]. A large retrospective study in patients with LC conducted by Reukenet al. found that women predominantly developed UTI and that the risk of infection was more strongly associated with age than with severity of liver disease measured using the MELD score [23]. A meta-analysis conducted to estimate the outcome of bacterial infection in cirrhosis found a 4-fold increased mortality in patients with LC, with pneumonia, SBP, and bacteremia being major contributors to increased mortality. One-month mortality in this population was estimated to be 30%, and another 30% of patients die within the year from infection [24]. Bruns et al. highlighted three major factors in determining mortality of bacterial infection in patients with LC, namely, severity of liver disease, concomitant renal failure, and the presence of antimicrobial resistance [5].

In our study, more than half the patients (*n* = 35, 53.8%) were found to be MDR. In Italy, Merli et al. found a similar percentage of MDR isolates in their patient cohort,whereas one Spanish study reported a lower percentage of MDR isolates (35%) [22]. Our results determined that *Enterococcus* spp. was the most common non-MDR pathogen (64.7%, *p* = 0.003), whereas *Enterobacteriaceae* were mainly MDR (81.1%, *p* = 0.001). In the present study concerning antibiotic resistance patterns, *Enterococcus* spp. showed high resistance rates to almost all antibiotics, reaching 100% for piperacillin-tazobactam, ertapenem, amikacin, ciprofloxacin, and levofloxacin among MDR strains, while *Enterobacteriaceae* isolates showed the highest resistance to penicillin, cephalosporins, and nitrofurantoin. However, in our study, HA-UTI due to *Enterococcus* spp. was found to be more common than in previously reported studies (52.3% vs 12–20%) [7, 8]. One reason for an increasing number of *Enterococcus* spp. isolates in Serbia might be its geographical location, as resistance patterns differ in relation to this factor. This observation of increased *Enterococcus* spp. prevalence was similar to a previous study that found most bacterial infections in patients with LC from central Europe were due to *Enterococcus* spp., whereas in southern Europe, ESBL-producing *Enterobacteriaceae* were found to be the main causes of bacterial infection [5].

Fernández et al., in a large prospective study of patients with LC who developed infection, found ESBL-producing *Enterobacteriaceae*, followed by *Pseudomonas aeruginosa*, methicillin-resistant *Staphylococcus aureus* (MRSA), and *Enterococcus faecium* to be the organisms most commonly associated with drug resistance [22]. Notably, the efficacy of empirical antibiotic treatment was decreased in patients with HA-UTI [22]. They concluded that, due to the increased use of broad-spectrum antibiotics, infections with MDR-Gram-negative organisms and *Enterococci* will continue to increase and remain a significant public health issue in future [22].

In the current study, we found statistically significant differences in regard to the distribution of MDR and non-MDR strains based on gram staining. The majority of Gram-negative isolates in our study were MDR, and Gram-positive bacteria were predominantly non-MDR isolates (*p* = 0.005). As expected, the rates of antibiotic resistance were significantly higher for MDR pathogens in comparison to non-MDR isolates for the majority of tested antibiotics (including ampicillin, and amoxicillin-clavulanic acids; meropenem, imipenem, and teicoplanin). Furthermore, resistance rates to cephalosporins were extremely high, ranging up to 100%, but the difference between MDR and non-MDR pathogens was clinically irrelevant due to extremely high resistance in both groups. The most effective antibiotics against *Enterococcus* spp. isolates, according to our results, were linezolid and vancomycin, with resistance rates of 25.0 and 41.7% for MDR, and 0 and 9.5% for non-MDR strains, respectively.

It has been demonstrated that failure of first-line empiric antibiotic therapy for bacterial infection in patients with LC is associated with increased mortality [5, 25]. According to data from southern and central Europe, ESBL-producing *Enterobacteriaceae* and *Enterococcus* spp. are associated with resistance to third-generation cephalosporins (TGC), which are currently recommended as the first empiric therapy for bacterial infection in patients with LC, especially in the setting of variceal bleeding, suspected SBP, or pneumonia [5]. A study by Campillo et al. showed that colonization with ESBL-producing *Enterobacteriaceae* doesnot correlate with the development of TGC-resistant infections in patients with LC [26]. Risk factors associated with TGC-resistant bacterial infections in patients with LC have been described. They include the following: HA infection, recent treatment with antibiotics (norfloxacin or β-lactam use within the previous three months), previous infection due to MDR bacteria, DM, and upper GI bleeding [5, 22]. However, there have been no randomized controlled trials to evaluate the effect of empiric therapy with carbapenems, tigecycline, or the addition of vancomycin (for enterococcus coverage) to ceftriaxone for empiric coverage inthe setting of HA in LC [5].

Similar to previously published studies [8] and as expected, patients with MDR UTI had a significantly higher empiric therapy failure rate (62.5%, *p* = 0.039). Our rates of empiric therapy failure were similar to those reported in an Italian study [8] where the authors identified empiric failure rates of 60% in an MDR group and 90% in the XDR strains. That study also concluded that failure of antimicrobial therapy led to deterioration in renal function, prolonged hospital stay, and higher in-hospital mortality [8].

According to our findings, older patient age is significantly associated with the development of MDR UTI, particularly in patients > 65. The etiology of LC was not associated with the presence of MDR strains. However, 85.7% of patients with an autoimmune etiology had a non-MDR UTI (*p* = 0.026). We did not find significant differences between patients with MDR and non-MDR UTI with respect to comorbidities, co-infections, presence of a urinary catheter, severity of liver disease, and outcomes. The presence of ascites did not differ between the groups. However, higher rates of encephalopathy, exposure to antibiotic within 7 days prior to the development of UTI, higher blood urea nitrogen, and serum ferritin were found in patients with UTI secondary to MDR strain.

D’Amico et al. proposed a classification of LC based on clinical stages, defined using criteria that occur throughout the natural history of the disease (varices, ascites, and variceal bleeding), adding infection as a further stage in the classification [27]. In accordance with a previous study, Dionigi et al. concluded that patients with LC who become infected have a greater risk of death even if they survive the acute episode of infection [28]. These findings suggest that infection represents a distinct prognostic stage of cirrhosis that affects survival irrespective of LC severity [29, 30].

In the current study, we used multivariable logistic regression to identify variables independently associated with the development of MDR UTI. We found that being ≥65 years old, empiric treatment with cephalosporin, and hepatic encephalopathy are independent predictors for the development of MDR UTIs. To the best of our knowledge, this is the first study that reports on the epidemiology of HA-UTI in patients with LC from Serbia and from the Balkans. In the absence of prospective studies on antimicrobial resistance patterns in the Balkans, we recommend clinicians implement five aspects of the Tarragona strategy, as follows: recognize individual patient risks, be familiar with the local epidemiology of bacterial strains and antimicrobial resistance, treat promptly and broadly, consider the site of infection, and re-evaluate therapy after 3 days [5].

### Study limitations

Our study uses retrospective data from only one tertiary care hospital from a small European country, limiting the external validity of our findings.

## Conclusion

Our study is the first epidemiological study concerning HA-UTI in Serbia and in the Balkans. We demonstrated that in the areas with a high prevalence of MDR bacterial strains, adherence to currently recommended empiric therapy is exceedingly difficult and is associated with high failure rates. The most common MDR pathogen among patients with LC and HA-UTI in our institution was *Enterococcus spp*. The overall resistance rate to third-generation cephalosporins was above 90%. Hence, the current guidelines may not be ideal for patients with decompensated LC who develop HA-UTIs.

We found that being ≥65 years old, the presence of hepatic encephalopathy, and preceding therapy with cephalosporins to be risk factors associated with the development of MDR infection. We suggest an individualized approach in selecting appropriate empiric antimicrobial therapy taking into the account local patterns of resistance and patient characteristics.

## Abbreviations

BUN: Blood urea nitrogen
CAIDS: Cirrhosis-associated immune dysfunction syndrome
CFU: Colony forming units
CI: Confidence interval
CLIF: Chronic liver failure
CLIF-C ADs: CLIF-consortium acute decompensation score
CLSI: Clinical and laboratory standards institute
DM: Diabetes mellitus
ECDC: European center for disease prevention and control
ESBL-E: Extended-spectrum beta-lactamase-producing *Enterobacteriaceae*
GI: Gastrointestinal
HA-UTI: Hospital-acquired urinary tract infection(s)
Ig: Immunoglobulins
IQR: Interquartile range
LC: Liver cirrhosis
MDR: Multi-drug resistant
MELD: Model of end-stage liver disease
MSSA: Methicillin-sensitive *Staphylococcus aureus*
MST: Microbial susceptibility testing
OR: Odds ratio
PDR: Pan-drug resistant
RE: Reticuloendothelial
ROC: Receiver operating characteristic
SBP: Spontaneous bacterial peritonitis
SIRS: Systemic inflammatory response syndrome
TGC: Third-generation cephalosporins
UTI: Urinary tract infection(s)
VRE: Vancomycin-resistant enterococcus
XDR: Extensively drug resistant
